# Event Centrality of Positive and Negative Autobiographical Memories in Older Adulthood

**DOI:** 10.1093/geroni/igab046.2646

**Published:** 2021-12-17

**Authors:** Justina Pociunaite, Tabea Wolf

**Affiliations:** Ulm University, Ulm, Baden-Wurttemberg, Germany

## Abstract

Centrality of an event (CE) is a characteristic denoting how important a life experience is to one’s identity. Usually, positive memories are more central than negative ones in the community samples. Nevertheless, there is emerging evidence showing substantial individual differences in how one perceives CE. Especially regarding age, one could expect pronounced differences due to age-related changes in personal goals. In this study, we investigated how older adults differ from young and middle-aged adults. Apart from age, we tested whether personality traits such as neuroticism and openness to experience influence the CE ratings among age groups. The sample comprised of 363 German participants, age ranging from 18 to 89 (M=49.57, SD=17.087), 67.2 % of the sample were women. Using multilevel analysis, we found the CE of positive memories to be higher in all age groups. The CE of positive events significantly differed for older adults compared to younger adults but not to the middle-aged group. With respect to personality, neuroticism had an impact only on the CE of negative memories in younger and middle-aged adults. For older adults, neither neuroticism, nor openness to experience had an impact on CE ratings. This shows that while older adults significantly differ from younger adults in the CE of positive memories, other individual differences characteristics do not have an impact on the way older adults perceive memories as central to their identity.

